# Modeling spatial interaction networks of the gut microbiota

**DOI:** 10.1080/19490976.2022.2106103

**Published:** 2022-08-03

**Authors:** Xiaocang Cao, Ang Dong, Guangbo Kang, Xiaoli Wang, Liyun Duan, Huixing Hou, Tianming Zhao, Shuang Wu, Xinjuan Liu, He Huang, Rongling Wu

**Affiliations:** a Department of Gastroenterology and Hepatology, Tianjin Medical University General Hospital, Tianjin Medical University, Tianjin, China; b Center for Computational Biology, College of Biological Sciences and Technology, Beijing Forestry University, Beijing, China; c School of Chemical Engineering and Technology, Frontiers Science Center for Synthetic Biology and Key Laboratory of Systems Bioengineering (Ministry of Education), Tianjin University, Tianjin, China; d Department of Gastroenterology, Beijing Chaoyang Hospital, Capital Medical University, Beijing, China; e Center for Statistical Genetics, Departments of Public Health Sciences and Statistics, The Pennsylvania State University, Hershey, PA, USA

**Keywords:** Gut microbiota, spatial biology, evolutionary game theory, microbial interaction network, quasi-dynamic ordinary differential equation

## Abstract

How the gut microbiota is organized across space is postulated to influence microbial succession and its mutualistic relationships with the host. The lack of dynamic or perturbed abundance data poses considerable challenges for characterizing the spatial pattern of microbial interactions. We integrate allometric scaling theory, evolutionary game theory, and prey-predator theory into a unified framework under which quasi-dynamic microbial networks can be inferred from static abundance data. We illustrate that such networks can capture the full properties of microbial interactions, including causality, the sign of the causality, strength, and feedback loop, and are dynamically adaptive along spatial gradients, and context-specific, characterizing variability between individuals and within the same individual across time and space. We design and conduct a gut microbiota study to validate the model, characterizing key spatial determinants of the microbial differences between ulcerative colitis and healthy controls. Our model provides a sophisticated means of unraveling a complete atlas of how microbial interactions vary across space and quantifying causal relationships between such spatial variability and change in health state.

## Introduction

Microorganisms residing in the human gut have direct and indirect impacts on health and disease.^1^ Disruptions to the community of gut microbes can contribute to the risk and severity of a number of medical conditions, such as obesity, cancer, and autism among others.^2–5^ While most studies focus on the determination of microbe-health relationships using homogenized samples, such as stools,^6–10^ there is growing appreciation for how the influence of microbes on host physiology is driven by the spatial heterogeneity of microbiota along the length of the digestive tract.^11–18^

Microbiota data collected at a series of gut locations are the key to chart the organizational and functional atlas of the microbial community across space,^18^ but this will critically rely on how these spatial data are modeled and analyzed. Trillions of microbes populate a narrow gut, forming the most complex and densest ecosystem on Earth. It is unlikely that microbes exert their influences independently of each other on host traits, rather they do so interactively through complex but well-orchestrated networks.^19–23^ More importantly, such interactions may occur among microorganisms not only at the same taxon level but also across taxonomic groups, given the co-occurrence of phylogenetically diverse microbes. As such, reconstructing large-scale microbial interaction networks across spatial gradients is a crucial choice to interrogate the elaborate crosstalk among gut microbes and between microbes and their hosts.

Here, we implement a computational model for inferring space-specific microbial interaction networks. Existing approaches reconstruct an overall microbial network from a large number of samples,^24^ failing to characterize sample-specific topological differences. Correlation-based appro-aches can estimate the strength of microbe-microbe interaction, but cannot infer the direction of the interaction.^25^ Bayesian networks can identify causality, but lack a capacity to find feedback cycles.^26^ High-density longitudinal data are powerful for reconstructing informative networks filled with bidirectional, signed, and weighted interactions, but such data are hardly available for the gut microbiota,^27^ especially at multiple locations of the gut. Our model that integrates elements of different disciplines can overcome all the above limitations to reconstruct informative, dynamic, omnidirectional, and personalized networks (idopNetwork) from static abundance data.^28–30^ The idopNetwork can reveal the position-dependent change of microbial networks and infer its spatially causal relationships with health state. As a proof of concept, we validate the usefulness of idopNetwork by designing and conducting a spatial mapping study of the gut microbiota. We identify key biogeographically-varying microbial interactions that distinguish diseased guts from healthy counterparts.

## Results

### Inferring ecologically functional microbial networks across gut space

**Figure 1.**
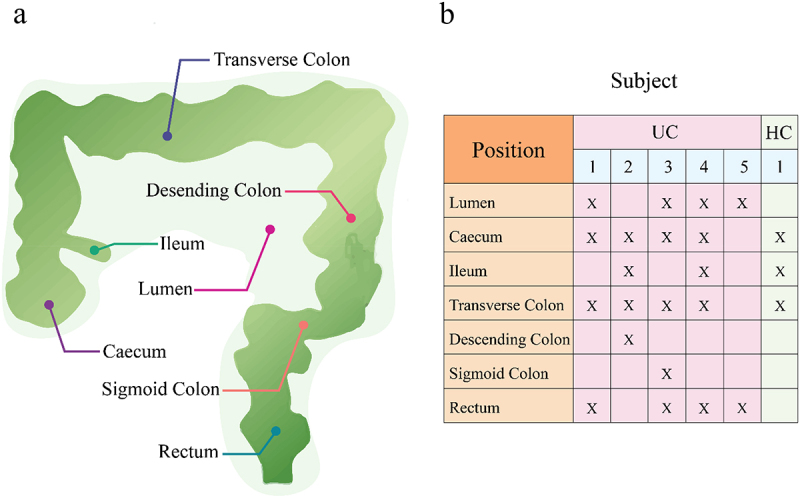
Sampling strategy of studying the spatial distribution of the gut microbiota. (a) Six sampled positions include rectum, sigmoid colon, descending colon, transverse colon, ileum, and cecum along the gut and lumen inside the gut. (b) Sample distribution of spatial microbiota from six guts, five infected by ulcerative colitis (UC) and one being a healthy control (HC), totalizing 23 independent samples.

**Figure 2.**
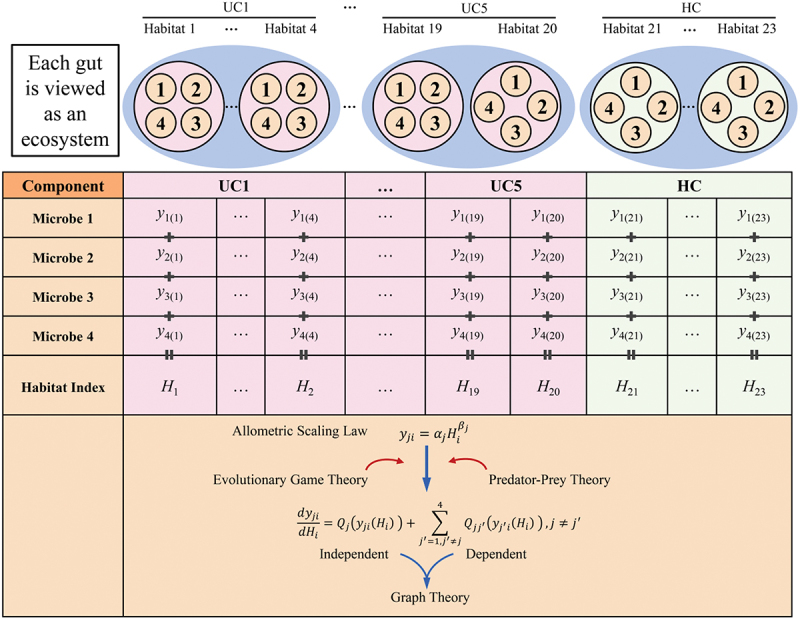
Workflow of idopNetwork reconstruction from spatial mapping data of the gut microbiota as collected from the study of Figure 1. Each gut can be viewed as an ecosystem in which different positions represent natural habitats of microbes. As a toy example, we assume four microbes residing at each gut position, whose abundance is monitored to form a data structure. By taking the sum of abundance of all microbes at each position, we calculate habitat index for this position. The power equation is used to model the allometric relationship between the abundance of individual microbes and habitat index, which establishes a foundation for converting static data into its quasi-dynamic representation crucial for the integration of evolutionary game theory and predator-prey theory into graph theory.

**Figure 3.**
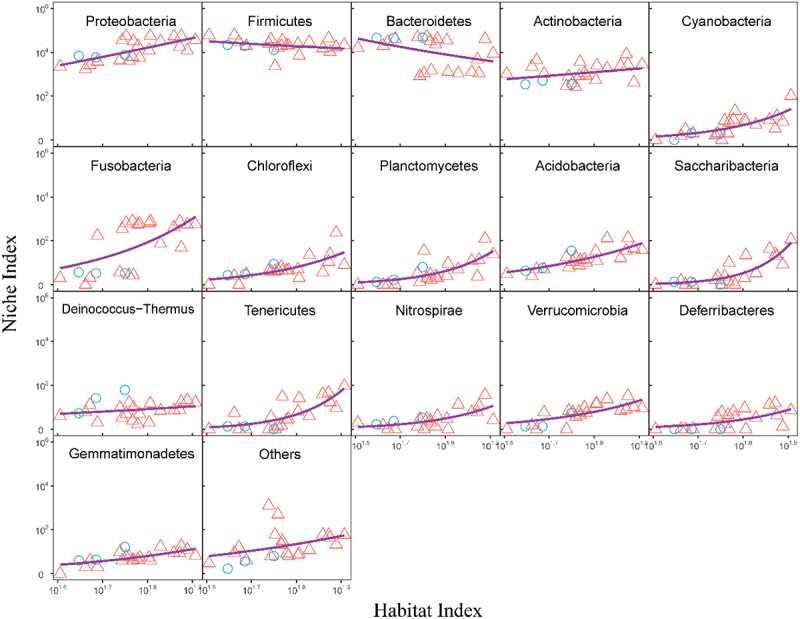
The fit of the power equation to the relationship between the abundance of individual phyla (niche index) and the total abundance of all phyla (habitat index) across UC (red triangles) and HC samples (blue circle).

We sample seven spatially different positions along and inside the guts from five patients infected with ulcerative colitis (UC) and one healthy control (HC) (Figure 1) and measure microbial abundance levels at different taxa from a total of 23 positions, i.e., habitats. The diagram in Figure 2 illustrates the workflow of how we reconstruct microbial networks from this spatial data. We identify the 16 most abundant phyla and the remaining phyla (attributed to the other) and plot the abundance level (i.e., niche index) of each phylum against habitat index (HI), defined as the summed abundance level of all microbes at a gut position. We find that niche index-HI change can be well fit by the power equation described in equation (1) (Figure 3). A majority of phyla increase their abundance with habitat index in an exponential manner (*R*^2^ = 0.80–0.90), although the slope of increase varies from phylum to phyla. Only two rich phyla, Firmicutes and Bacteroidetes, follow a reverse pattern of change, i.e., decreasing exponentially with habitat index. We plot the niche index of each species against habitat index and find that their relationship can be similarly fitted by the power equation, but with the slope depending on species (**Fig. S1**). Taken together, the power equation can not only explain the part-whole relationship of individual microbes at various taxonomic levels with the total microbes, but also provides a means of modeling microbial diversity and interactions across spaces.

**Figure 4.**
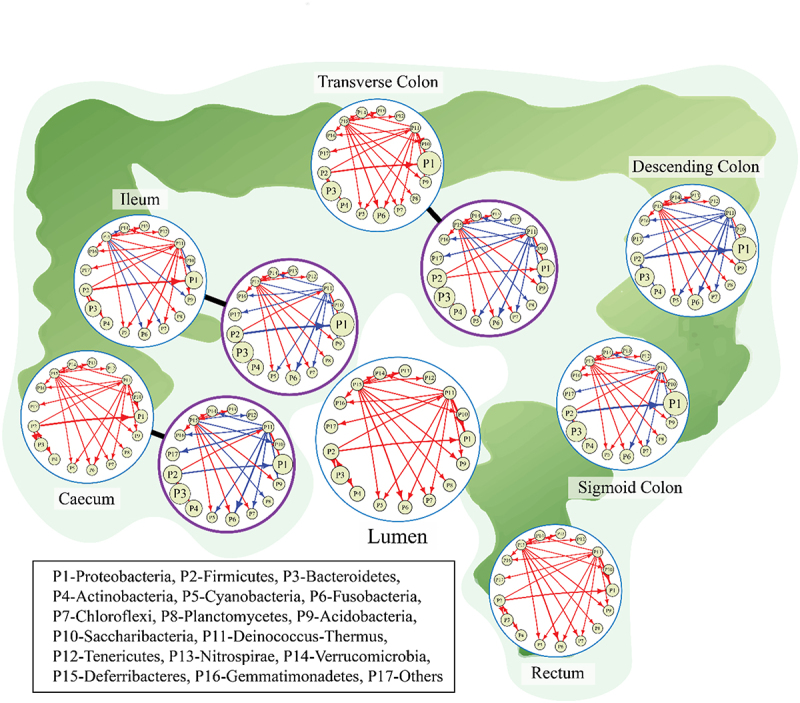
Spatial microbial networks at the phylum level along and inside the gut. Arrow lines with warm and cold colors represent commensalism and amensalism, respectively, with the thickness of lines proportional to the strength of microbe-microbe interactions.

In the Materials and methods section, we describe the derivation of the idopNetwork model by building quasi-dynamic ordinary differential equations (qdODE) based on niche index-HI relationship (Figure 2). The qdODE decompose the observed abundance level of a microbe into its independent component due to its own capacity and dependent component resulting from the influence of other microbes, as shown by equations (2) and (7). We use the independent and dependent abundance components estimated by an optimization technique to reconstruct spatially-varying idopNetworks at the phylum level for UC and HC groups (Figure 4). The topological organization of microbial networks displays both similarities and differences among gut positions. For example, Deferribacteres acts as a leader (i.e., those with more and stronger outgoing links than other microbes) that exerts influences on Cyanobacteria, Fusobacteria, Chloroflexi, Planctomycetes, and Acidobacteria in all networks, regardless of their gut positions and gut health states. There is also gut position-dependent variability not only expressed in total microbial abundance and microbial composition (Figure 3), but also in the topological structure and organization of microbe-microbe interactions (Figure 4). Position-dependent networks of the UC group can be clearly classified into two types; one involving lumen, rectum, transverse colon, ileum and cecum networks and the second including sigmoid colon and descending colon networks. In the second type, Firmicutes and Deinococcus-Thermus bring strong amensalism to bear on Proteobacteria and Fusobacteria, respectively, whereas in the first type, the former is commensalistic toward the latter. Detailed position-dependent differences can be observed in network organization from the same type. In the first type, the strength of this commensalism decreases from the cecum and ileum networks to the transverse colon network to the rectum and lumen networks. Deferribacteres is commensalistic for Fusobacteria in the lumen, rectum, transverse colon, and cecum networks, but this relationship changes as amensalism in the ileum network. In the second type, compared to the sigmoid colon network, the descending colon network is encoded by amensalism at a higher frequency.

Only three positions, transverse colon, ileum, and cecum, were sampled for the HC group, and these positions display distinct network topologies (Figure 4). Each of these three HC networks is tremendously different from the UC network at the same position. In general, the HC networks are well mixed by commensalism and amensalism, whereas the UC networks tend to be dominated by commensalism, suggesting that a healthy network can better balance different types of microbes than a diseased network. At the cecum and ileum positions, Firmicutes establishes a commensalistic relationship toward Proteobacteria in the UC networks, but an amensalistic relationship between these two phyla is detected in the HC networks. In the transverse colon network, although commensalism occurs from Firmicutes to Proteobacteria in both UC and HC groups, the strength of this interaction is larger in the former than in the latter. Also, Deinococcus-Thermus is commensalistic toward Fusobacteria in the UC networks, but this relationship is amensalistic in the HC networks. Taken together, the strength, pattern and architecture of microbial interactions vary spatially across the biogeographic positions of the gut, with the degree of variation depending on healthy state. Network analysis on the three commonly measured positions suggests that healthy networks display a greater position-dependent variability than diseased networks. A series of spatially reconstructed networks can more precisely characterize key microbial interactions that interrogate why and how a healthy state becomes unhealthy.

**Figure 5.**
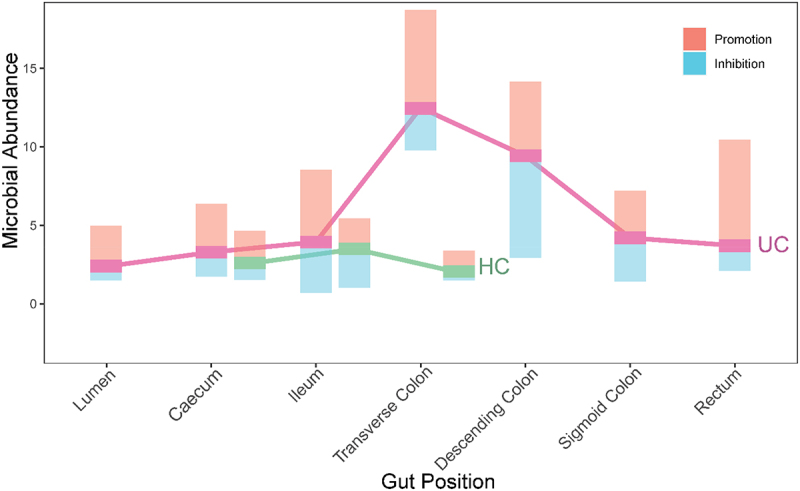
Microbial abundance of all phyla at different gut positions, decomposed into the independent component (purple bar for UC, green bar for HC), positive dependent component (due to promotion, warm bar), and negative dependent component (due to inhibition, cold bar).

Our qdODE model decomposes the observed abundance level of a microbe into its independent component (expressed when this microbe is socially in isolation) and dependent component (due to the accumulated (positive and negative) influence of other microbes on this microbe) at each gut position. This decomposition is illustrated in Fig. S2, where the independent and dependent abundance of each phylum are characterized across samples. Figure 5 reveals the position-dependent total amounts of independent abundance, positive dependent (promotion) abundance and negative dependent (inhibition) abundance over all microbes at the phylum level. For UC guts, lumen, cecum, ileum, sigmoid colon, and rectum has a similar amount of independent abundance, which is much lower than that at transverse colon and descending colon. Among three commonly measured positions, cecum and ileum are similar in independent abundance between UC and HC guts, whereas the independent abundance of transverse colon is strikingly larger in the UC than HC guts. This suggests that the intrinsic capacity of microbes to express themselves in transverse colon is a determinant of the health state of a gut. The amounts of positive dependent abundance (via promotion) and negative dependent abundance (via inhibition) substantially vary among gut positions. Some positions, such as ileum, transverse colon, and rectum, have a large amount of positive dependent abundance, whereas some positions, like descending colon, are dominated by negative dependent abundance (Figure 5). At transverse colon, both positive and negative dependent abundance are much richer for UC guts than HC guts, suggesting that the strength and pattern of microbial interaction at transverse colon are a determinant of gut health state. Taken together, when healthy guts get infected by ulcerative colitis, the capacity of microbes to be independently expressed increases at certain positions, i.e., transverse colon and descending colon. Also, the total strength of microbial interaction and the relative strength of cooperation and competition are associated with the shift of health state from healthy to diseased and vice versa.

### Reconstructing multilayer and multiplex microbial networks

Microbial networks may occur and function at different taxonomic levels, with larger sizes at lower than higher levels. Modularity theory suggests that a robust and stable large-scale network would be divided into distinct network communities within which entities are more functionally correlated with each other than with those from other modules.^31,31^ Thus, when we attempt to reconstruct a microbial network from a large number of microbes, we can break it down into sparsely interconnected network communities. Wu and Jiang^30^ proposed a bottom-up approach for identifying network communities by classifying all entities into different modules according to their similarity of sample-dependent variability. As shown by Figure 2, the abundance of a microbe changes with habitat index following the power law. We implement the power equation into Kim et al.^31^ functional clustering to detect different microbial modules (see the Method).

The mean values of abundance for all microbes within modules are used to reconstruct the coarse-grained module-module idopNetwork or the top-layer microbial network. Microbes within each module form multiple fine-grained microbial networks or bottom-layer microbial networks. The dimension reduction of data by clustering increases statistical and computational efficiency in inferring coarse- and fine-grained networks. However, if the number of microbes within a module is still too large to be used for network reconstruction, we can implement functional clustering to classify this module into distinct submodules and reconstruct cross-submodule networks. If needed, a submodule can be further classified into different sub-submodules and this process repeats until a trackable number of microbes is obtained. In the end, we reconstruct multilayer microbial networks at different levels of classification. If microbes at a higher classification level interact with those at a lower level, multiplex microbial networks can be reconstructed.

**Figure 6.**
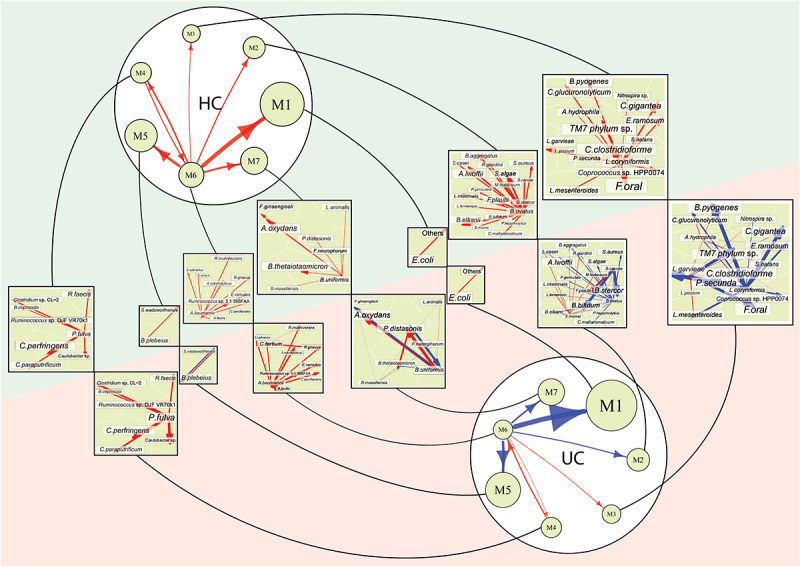
Two-layer microbial networks at the species level for HC and UC groups. At the top level of the network is the 7-node inter-module (coarse-grained) network and at the bottom level are seven interspecific (fine-grained) subnetworks, visualized by Voronio treemaps. Arrow lines with warm and cold colors represent commensalism and amensalism, respectively, with the thickness of lines proportional to the strength of microbe-microbe interactions.

In our spatial mapping study, we identify 65 species, which are classified into seven distinct modules M1-M7 by functional clustering (Fig. S3). Each module contains a different number of species, forming a network community. We reconstruct a seven-node coarse-grained microbial network from modules and seven fine-grained microbial networks from species of the same modules, forming a 2-layer microbial network between and within modules (Figure 6). Such a 2-layer network is inferred for UC and HC groups, respectively. The first-layer network is structurally similar but organizationally different between the two groups. Module M6, as a leader, exerts a strong commensalism to modules M1 and M5 in HC guts, but this relationship becomes amensalistic in UC guts.

M1 and M5 are highly abundant, but each is only composed of two species. In M1, *Escherichia coli* and those unidentified species establish a commensalistic relationship in both HC and UC groups, but in M5 *Bacteroides plebelus* prompts *Suttereslla wadsw* in the HC group, but the latter turns to inhibit the former in the UC group (Figure 6). Considerable differences in both structure and organization are observed for the second-layer networks of the other five modules. Compared to the UC group, the second-layer networks of M2, M3, and M7 contains more amensalistic links than those in the HC group, further suggesting that microbial competition may be a cause of ulcerative colitis. For example, in the second-layer network of M2, *Bacteroides ovatus* is a positive strong leader that promotes many other species in HC guts, but its leadership becomes negative at a reduced strength in UC guts. Also, *Bacteroides caccae* is activated in UC guts, exerting strong commensalism for *Flavonifractor plautii* and strong amensalism for *Bifidobacterium bifidum* and *Bacteroides sterco*.

More remarkably, the second-layer network of M3 produces structural and organizational changes from HC to UC groups (Figure 6). *Lactobacillus coryniformis* is a dominant leader that positively impact many other species in the HC group, but it turns to exert strong negative impacts on the same species in the UC group. *Lactococcus piscium* that coexists peacefully with its partners in HC guts becomes aggressive toward *Firmicutes oral* and *phylum sp. Oral* in UC guts. M4 and M6 are filled of commensalism in both HC and UC networks, but the strength of commensalism notably differs between two types of networks. Stronger commensalism exerted by particular species is observed in UC than HC groups. Taken together, multilayer microbial interaction networks provide a detailed roadmap of how each microbial species interacts with every other species through cooperation or competition to determine the change of guts from healthy to unhealthy states.

### Spatial tracing of multilayer networks

**Figure 7.**
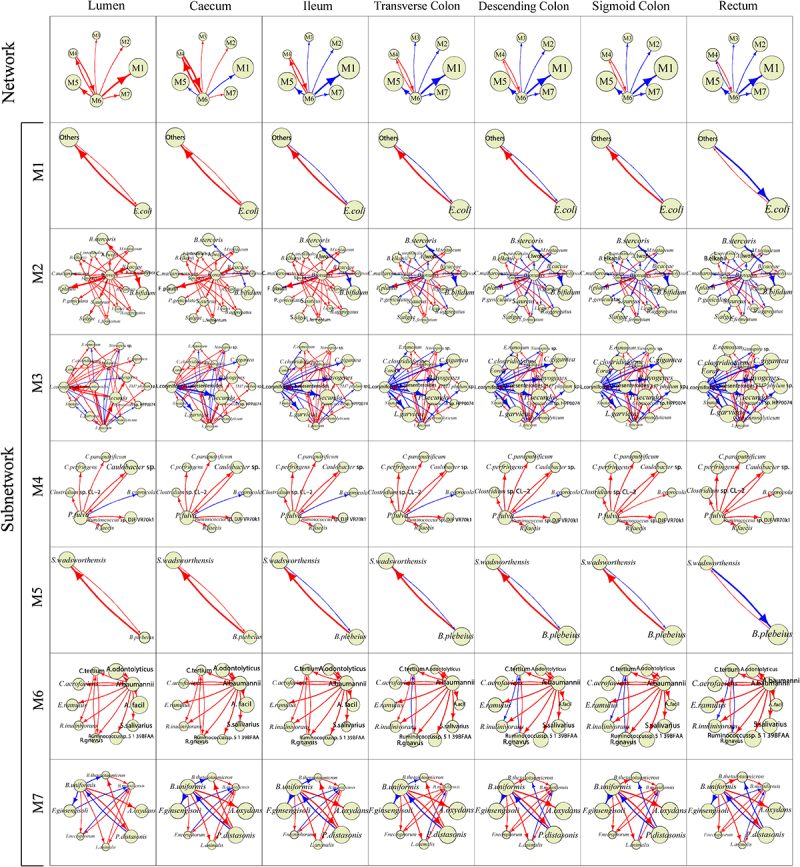
Two-layer microbial networks at the species level over different gut positions for the UC group. For each position, at the top level of the network is the 7-node inter-module (coarse-grained) network and at the bottom level are seven interspecific (fine-grained) subnetworks, visualized by Voronio treemaps. Arrow lines with warm and cold colors represent commensalism and amensalism, respectively, with the thickness of lines proportional to the strength of microbe-microbe interactions.

**Figure 8.**
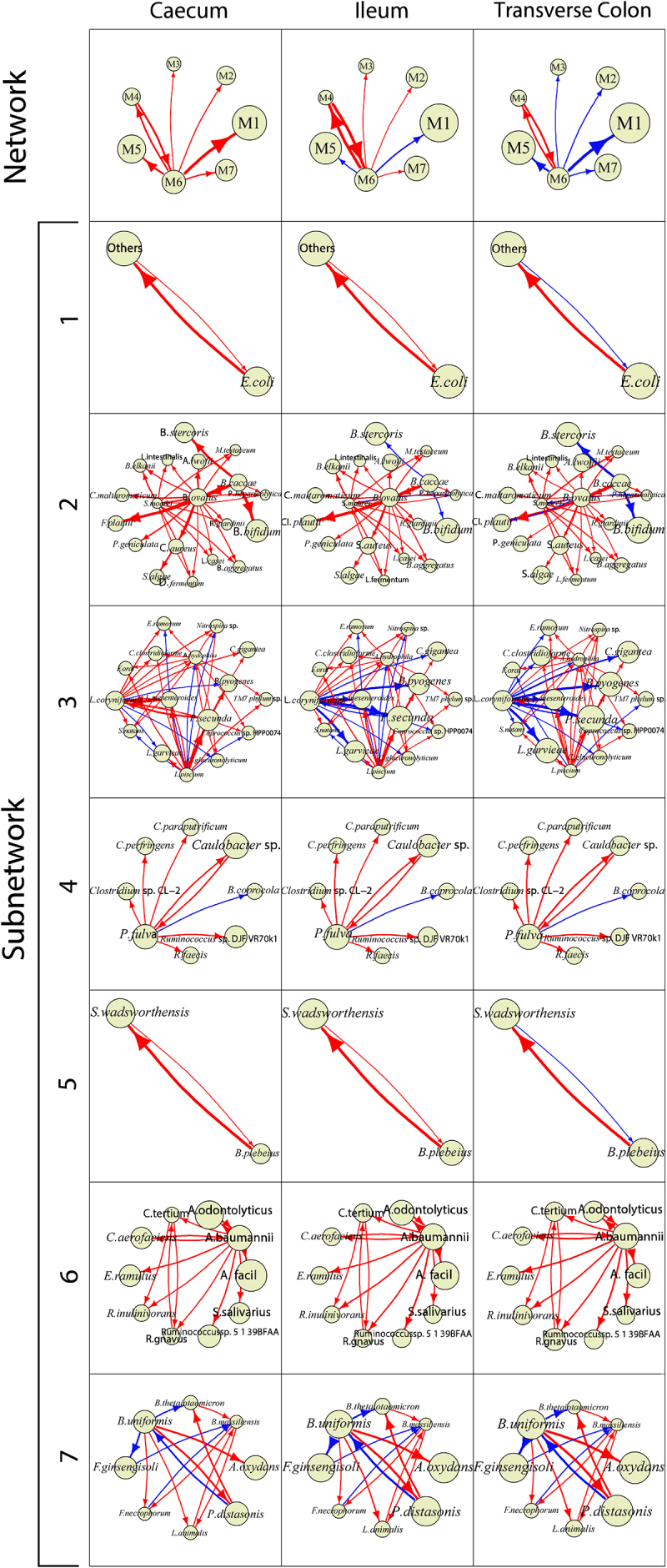
Two-layer microbial networks at the species level over three different gut positions for the HC group. For each position, at the top level of the network is the 7-node inter-module (coarse-grained) network and at the bottom level are seven interspecific (fine-grained) subnetworks, visualized by Voronio treemaps. Arrow lines with warm and cold colors represent commensalism and amensalism, respectively, with the thickness of lines proportional to the strength of microbe-microbe interactions.

We reconstruct multilayer networks at each position from which we can trace how coarse- and fine-grained networks vary along biogeographical gradients (Figures 7, 8). For the UC group, coarse-grained networks are structurally very similar over seven sampled positions, except for the link from modules M6 to M1, which is commensalistic at lumen, cecum, and ileum but amensalistic at transverse colon, descending colon, sigmoid colon, and rectum (Figure 7). Fine-grained networks also vary among gut positions, but with the degree of this difference depending on modules. Network communities for M4 and M6 exhibit a slight increase of commensalism strength from lumen to rectum, but those for M1, M2, M3, M5, and M7 differ dramatically among positions. In those network communities, some species-species links change from commensalism to amensalism. For example, in M5, *Bacteroides plebeius* and *Sutterella wadsworthesis* are mutualistic to each other at lumen, cecum, and ileum, but become increasingly parasitic from transverse colon to descending colon to sigmoid colon to rectum. Similarly, in M3, *Lactobacillus coryniformis* is commensalistic to *Lactococcus garvieae* at lumen and cecum, but this relationship becomes amensalistic with an increasing strength from ileum to transverse colon to descending colon to sigmoid colon to rectum.

The three gut positions sampled for the HC group also exhibit differences in both coarse- and fine-grained networks, although the extent of such differences is module-dependent (Figure 8). In the coarse-grained network, leader M6ʹs commensalism toward M1 and M5 at cecum and ileum becomes amensalism at transverse colon, but the internal workings within M6, M1, and M5 little vary among three positions. Pronounced differences in the sign and strength of certain interspecific interaction are observed in fine-grained networks of M2, M3, and M7. For example, in M2, *Bacteroides caccae* exerts commensalism at cecum and ileum but amensalism at transverse colon for *Bacteroides stercoris* and *Bifidobacterium bifidum*. In M7, *Bacteroides uniformis* is commensalistic toward *Flavisolibacter ginsengisoli* at cecum, but this commensalism changes as amensalism at ileum and transverse colon.

The two-layer networks provide a detailed atlas of interspecific interactions that determine differences between UC and HC groups (Figure 7 vs. Figure 8). Although most pairwise interactions are consistent in sign between the two groups, there are a few certain interactions that change their sign from HC to UC guts. In general, UC networks contain more amensalistic interactions than the HC networks, suggesting that microbes tend to turn to be competitive when the guts are infected by ulcerative colitis. For example, in M7, *Bacteroides uniformis* at cecum is commensalistic toward *Flavisolibacter ginsengisoli* in the HC group, but becomes amensalistic toward the same species in the UC group. In addition, for some pairs of species, the strength of their interactions may change from HC to UC at the same position. All suggest that certain interspecific interactions may be used as a biomarker to assess health risk.

## Discussion

The network model described in this article adds a previously neglected dimension to fully capture the topology of the vast complexity of the gut microbiota. This additional dimension is the spatial distribution of microbes among physically, chemically and immunologically distinct niches in the gut.^12,32,^ The biogeography of bacteria in the gut is regulated by nutrient selection and immune activation and, meanwhile, it determines the microbial compositions and heterogeneity along the longitudinal axis of the intestines. However, a precise characterization of microbial interaction networks along gut positions is challenged by unavailability of high-density dynamic or perturbed abundance data collected at each position. In this article, we present a spatial model to disentangle this challenge by extracting dynamic snapshots from static data.

The capacity of the new model to dynamize networks in space with no need of dynamic data results from the seamless integration of multiple disciplines through the implementation of high-dimensional statistical theory. From a dynamic perspective, evolutionary game theory explains the overall abundance of a microbe to be due to its independent component (reflecting how much a microbe can grow in isolation due to its intrinsic capacity) and dependent component (describing the amount of microbial growth due to influence by other co-existing members). The dynamic formulation of evolutionary game theory using static data is achieved by converting them into their quasi-dynamic representation via allometric scaling theory. By introducing predator-prey theory, we construct a system of qdODE to quantify the independent and dependent components for each microbe.^28,29,30^ The estimated independent and dependent components by an optimization technique are coded into graphs, producing one of the most advanced networks – idopNetwork. As compared to the most commonly used Dynamic Bayesian Networks (DBNs) which can identify the causality of interactions from evenly-spaced dynamic data, idopNetwork can extract and capture full properties of microbial interaction networks (including bidirectionality, sign, weight, and feedback loop) from any data domain with any measurement schedule. Through extensive computer simulation studies, idopNetworks were found to perform much better than DBNs in terms of the power and false positive rate of interaction detection.^32^ In particular, idopNetworks provide a visualized platform to trace how microbial interaction architecture changes from one location to the next and how these changes impact the reciprocal shift between healthy state to diseased state.

As a proof of concept, we apply idopNetwork to reveal how microbial network structure and organization vary along biogeographical gradients of the gut using the gut microbiota data from a spatial mapping study including ulcerative colitis-infected patients and healthy controls. idopNetwork characterize a dramatic change in network organization (the sign and strength of interactions) from one position to next along and inside the gut (Figures 3, 5, 7, 8). Such an organizational change is much more pronounced in guts inflected by ulcerative colitis than healthy guts, suggesting that the position-dependent change of interaction strength and sign may be a driver of gut shift from a healthy state to an unhealthy state.

An increasing number of studies have showed a great potential to control many diseases by changing the ecological equilibrium of microbes in the gut,^33,34,35^ but existing approaches can only treat the whole gut as a mixture, without taking into account position-dependent variability in the gut microbiota. The idopNetwork model can discern spatial changes of microbial interactions and identify the precise gut positions at which microbial functions are most tightly associated with the disease. Spatial data analysis shows that difference between ulcerative colitis guts and healthy guts lies in the shift of ecological interactions between specific microbes from commensalism-dominated cooperation to amensalism-dominant competition at certain gut positions. Phylum Planctomycetes exerts strong commensalism for phylum Verrucomicrobia at transverse colon and cecum in healthy guts, but this commensalism becomes strong amensalism when the guts are inflected by ulcerative colitis. In clinical practice, by shifting Planctomycetes-Verrucomicrobia amensalism back to Planctomycetes-Verrucomicrobia commensalism at these gut positions via medical interventions, ulcerative colitis may be controlled and eliminated.

The idopNetworks can be reconstructed at any phylogenetic level, including a web of crosstalk among bacteria, fungi, and viruses and networks at any taxonomic level for the same type of microbes. While microbial networks at a higher level of taxon, such as phylum, can generalize a general rule of thumb behind microbial community assembly, those at a lower level, such as species, strain, or even genotype, can precisely characterize the detailed roadmap of how individual microbes interact with each other spatially to determine host health. However, a low taxonomic level may include an enormous number of operational taxonomic units (OTU), which makes it computationally infeasible to reconstruct a large-scale network. To circumvent this issue, we implement power equation-functional clustering to classify all OTUs into distinct size-reduced modules according to developmental modularity theory. In network science, this procedure is a bottom-up approach that breaks down any large-scale network into distinct network communities or subnetworks.^30^ We use this approach to classify 65 species into seven modules and reconstruct a two-layer network involving all species, at a higher layer of which is a 7-node module-module network and a lower layer of which are seven species-species networks within modules. Two-layer networks display gut position-dependent variability, healthy state-dependent variability, and position-state interaction variability in topological organization. The shift of commensalism to amensalism between *Bacteroides uniformis* and *Flavisolibacter ginsengisoli* at cecum (within the subnetworks of module M7) could be a driver of healthy guts that turn to ulcerative colitis-infected (Figure 7 vs. Figure 8). Yet, at ileum and transverse colon, these two species consistently maintain an amensalistic relationship, regardless of health state. Thus, *Bacteroides uniformis-Flavisolibacter ginsengisoli* interaction at these two positions cannot be used to distinguish between healthy guts and ulcerative colitis-infected guts.

Although idopNetworks have many advantages, the above results from our spatial mapping study of the gut microbiota should be interpreted with caution. First, the sample size (23 positions) used may be insufficient to make a rigorous inference. Computer simulation shows that at least 20 samples are required to obtain reasonable power for interaction detection, but increasing sample sizes, such as 50 to 100, are necessary if the measurements of microbial abundance contain a certain level of noises.^28,30^ Second, as a proof of concept, our data only contain one healthy control. At least three biological replicates for the control are required to reasonably infer about microbial differences between diseased and health groups. Third, the impact of the gut microbiota on host health is determined by genetic, demographic, and environmental factors.^36^ The utility of idopNetwork can be best justified only after it is implemented into a genome-wide association studies aimed at characterizing the combined effects of various genetic and environmental factors on the microbiota-health interplay. Despite these limitations, the application of idopNetwork in this study provides a starting point for deciphering how ecological interactions transit along biogeographical locations to shape the resilience, stability and diversity of microbial ecosystems and human health from a large-scale data domain. Furthermore, idopNetwork can be brought into play to unleash a broader microbial community assembly including oral cavity, esophagus, and vaginal microhabitats.

## Conclusions

The spatial variation of microbes determines how the gut microbiota impacts human health. This impact can be better revealed by inferring spatially dynamic interaction networks. In this article, we develop a model for tracing network dynamics along space with no need of dynamic data. Statistical analysis of microbial data according to this model includes four steps: (i) fitting of the power equation to the abundance of individual microbes over habitat index, (ii) LASSO-based variable selection implemented to choose a small set of the most significant microbes that are linked with a focal microbe, (iii) network reconstruction based on qdODE, and (iv) functional clustering of all microbes into distinct modules based on their pattern of microbial abundance. The last step allows large-scale multilayer sparse microbial interactome networks across gut space to be reconstructed. Our model can discern interaction changes of any microbes from healthy to unhealthy guts to gain new insight into microbial impact on human health and disease.

## Materials and methods

### Allometric scaling law

Consider a spatial mapping study of the gut microbiota involving multiple subjects. For each subject, the abundance of microbes at different taxa is monitored at seven distinct positions, i.e., cecum, ileum, transverse colon, descending colon, sigmoid colon, and rectum along the gut, and lumen inside the gut (Figure 1). If a gut is viewed as an ecosystem, different spatial positions inside and along the gut form multiple ecological habitats. Let *y_jkv_* denote the abundance of microbe *j* at position *k* from gut *v*. Note that a microbe considered here may represent an OTU at any given level, such as individual, strain, species, genus, family, order, class, or phylum. For habitat *k* from gut *v*, the total amount of abundance of all colonizing microbes (say *m*), i.e., $H_{kv}=\overset{m}{\underset{j=1}{\sum }}y_{jkv}$, reflects its overall capacity to carry and feed the microbes with essential resources for their survival and propagation, which is defined as the habitat index. From an ecological perspective, the habitat index is determined by a mixture of factors including host genes, diet types and life style,^36,37^ a concept similar to the environmental index coined to describe the overall quality of site in terms of the accumulative growth of all plants.^38,39^ How much a given microbe within a habitat is expressed is the confounding consequence of its intrinsic capacity and its interactions with all possible biotic and abiotic factors. We thus define the abundance of a microbe in a habitat as the niche index.^37,^

A habitat forms a microbial community within the gut, with habitat index ($H_{kv}$) describing the whole community behavior and niche indices ($y_{jkv}$) describing components of the community. From a physical perspective, habitat index and niche index establish a part-whole relationship, which can be described by the power function according to allometric scaling theory.^40^ Let *n* denote the total number of habitats from all subjects (guts). Thus, for a given habitat *i* (*i* = 1, …, *n*), this part-whole relationship can be mathematically expressed as

(1) $$y_{ji}=\alpha_{j}H_{i}^{\beta_{j}}$$

where *β_j_* is the scaling exponent and *α_j_* is an intercept constant of microbe *j*, which together determine the scaling shape of individual microbes with habitat index. Previous studies have shown that this power equation fits microbial abundance at any taxonomic levels across habitats.^30^

### Quasi-dynamic evolutionary game theory

Game theory states that a rational player strives to maximize its payoff by choosing an optimal strategy in response to the strategies of other players until the Nash equilibrium is reached.^41,42^ As can be seen, such a strategy choice is not arbitrary, but rather includes a rational judgment based on a player’s accrued knowledge of the environment affected by other players. However, it seems unreasonable to assume the rationality of microbes in making their decision. We introduce evolutionary game theory, the combined theory of game theory and evolutionary biology,^43,^ which uses the concept of an evolutionarily stable strategy (ESS) to refine the Nash equilibrium. In an evolving population, any strategy used by a player to maximize its payoff would be constrained by strategies of other players that also strive to maximize their own payoffs and, ultimately, this process through natural selection would optimize the structure and organization of the population, making it reach the maxima of its overall payoff.^44,^ The dynamic modeling of evolutionary game theory does not specify any individual ESS, but it bears all of the ESS that change in the population,^45,46^ in which case the rationality assumption is relaxed.

Although there is no time dimension in our microbiota mapping study, allometric scaling law, described by equation (1), provides a bridge to dynamically model evolutionary game theory by deriving a system of generalized nonlinear predator-prey qdODE with the time derivative replaced by the habitat index derivative.^28,29,30^ Each qdODE specifies the overall abundance of a microbe that is determined by its own “strategy” and the “strategies” of its interacting counterparts. The qdODE that quantify the nLV system are expressed as

(2) $$\frac{dy_{ji}}{dH_{i}}=Q_{j}\left(y_{ji}\left(H_{i}\right);\Theta_{j}\right)+\overset{m}{\underset{j{\;}^{\prime }=1,j^{\prime }\neq j}{\sum }}Q_{jj{\;}^{\prime }}\left(y_{j{\;}^{\prime }i}\left(H_{i}\right);\Theta_{jj{\;}^{\prime }}\right),i=1,\ldots ,n;j=1,\ldots ,m$$

where the abundance of any microbe *j* expressed at habitat *i* is decomposed into the *independent* component ($Q_{j}\left(\cdot \right)$) and *dependent* components ($Q_{jj{\;}^{\prime }}\left(\cdot \right)$). The independent component of microbe *j* is determined by its intrinsic capacity, which can be fully expressed when it is in isolation, whereas the dependent component of microbe *j* is the aggregated effect of influences on it by all other microbe *j*′ (*j*′ = 1, …, *j* – 1, *j* + 1, …, *m*). Functions, $Q_{j}\left(\cdot \right)$ and $Q_{jj{\;}^{\prime }}\left(\cdot \right)$, may not have explicit forms, but they can be smoothened by a nonparametric approach, such as B-spline or Legendre orthogonal polynomials (LOP),^47,48^ where $\Theta_{j}$ and $\Theta_{jj{\;}^{\prime }}$ are the unknown parameters that specify the nonparametric curves. We code the estimates of $Q_{j}\left(\cdot \right)$ and $Q_{jj{\;}^{\prime }}\left(\cdot \right)$ as nodes and edges, respectively, into quantitative networks as a tool to characterize the structure and organization of microbial community assembly along the gut.

### Sparse microbial networks

In practice, the number of microbes for network reconstruction may be very large, thus if the abundance of each microbe involves the effects of all other microbes, ODEs (2) will quickly become intractable. In biology, it is unlikely that each microbe performs an interaction with every other microbe in the community because a fully connected network is not helpful for the organism to maintain its robustness and stability in response to random perturbations.^49,50,51^ Therefore, even if there are a number of microbes within a habitat, interaction networks they constitute are likely to be sparse. Sparsely connected networks are consistent with Dunbar’s laws. In modeling social networks of non-human primates, Dunbar^52,^ found that there is a limit to the number of relationships within a network an individual can stably maintain because of a limited size of its neocortex. Dunbar’s finding on the limit of social relationships, now known as Dunbar’s law, has been considered as a general argument for network reconstruction in a wide range of physical and life sciences.^53,^

To choose a subset of the most significant microbes that interact with a given microbe, we formulate a multiple regression model that regresses the abundance of the microbe (response) on the abundance of all other microbes (predictors) across habitats. A regularization-based variable selection approach, such as LASSO^54,^ and its variants,^55,56,57^ is implemented to shrink the dimension of links possibly owned by a microbe.

**Regression model**. We consider each gut position from each individual (healthy or unhealthy) as a sample. A sample represents a microbial community assembly that contain *m* microbes. Let **y***_j_ *= (*y_j_*(*W*_1_), …, *y_j_*(*W_n_*)) denote a vector of observed abundance values for microbe *j* (*j* = 1, …, *m*) in *n* samples. Based on the structure of qdODE in equation (2), this microbe’s habitat index-varying abundance level can be described by a multiple regression model, expressed as

(3) $$y_{j}\left(W_{i}\right)=G_{j}\left(y_{j}\left(W_{i}\right):\Theta_{j}\right)+\overset{m}{\underset{j{\;}^{\prime }=1,j^{\prime }\neq j}{\sum }}G_{jj{\;}^{\prime }}\left(y_{j{\;}^{\prime }i}\left(W_{i}\right):\Theta_{jj{\;}^{\prime }}\right)+e_{j}\left(W_{i}\right)$$

(4) $$=a_{j}\left(W_{i}\right)+X_{j}^{T}b_{j}\left(W_{i}\right)+e_{j}\left(W_{i}\right).$$

In equation (3), and $G_{jj{\;}^{\prime }}\left(\;\right)$ are the habitat index-varying independent and dependent abundance of microbe *j*, whose derivatives are $Q_{j}\left(\;\right)$ and $Q_{jj{\;}^{\prime }}\left(\;\right)$ of equation (1), respectively, ^and^ $e_{j}\left(W_{i}\right)$ in equation (4) is the residual error of microbe *j* at sample *i*, obeying a multivariate normal distribution with mean vector **0** and sample-dependent covariance matrix for microbe *j*. We assume that the residual errors of microbial abundance are independent among samples so that $\sum_{j}$ is structured as $\sum_{j}\sigma_{j}^{2}I_{n}$ where $\sigma_{j}^{2}$ is the residual variance of microbe *j* at the same sample and **I***_n_* is the identity matrix. In equation (4), we have $a_{j}\left(W_{i}\right)=$ $G\left(\;\right)$ and $X_{j}^{T}b_{j}\left(W_{i}\right)=\overset{m}{\underset{j{\;}^{\prime }=1,j^{\prime }\neq j}{\sum }}G_{jj{\;}^{\prime }}\left(\;\right)$, where $X_{j}^{T}$ is the vector containing *m* – 1 ones and **b***_j_*(*W_i_*) = (*b_j_*_1_(*W_i_*), …, *b_jm_*(*W_i_*)) is a vector of the dependent value of microbe *j* determined by all microbes, except for *j*.

**Group LASSO**. LASSO is particularly powerful for the penalty regression analysis of a response on an extremely large number of predictors across a much smaller size of samples. Considering a focal microbe *j* as a response, we use nonparametric independent parameters **a***_j_* to fit its $G_{j}\left(\;\right)$. As predictors, (*m* – 1) microbes contribute to microbe *j*’s dependent component through unknown nonparametric dependent parameters **β***_j_*** = **(**β***_j_*_1_, …, **β***_j_*_(*j*–1)_, **β***_i_*_(*j*+1), …_, **β***_jm_*). Thus, we have *m* – 1 groups of dependent parameters that reflects the influence of other microbes on the focal microbe. We implemented group LASSO^55^ to select those nonzero groups. The group LASSO estimators of dependent parameters, denoted as **= **(**β***_j_*_1_, …, **β***_j_*_(*j*–1)_, **β***_j_*_(*j*+1), …_, $\beta \;jd_{i}$), where *d_j_ m* is the number of the most significant microbes that interact with microbe *j*. It can be obtained by minimizing the following penalized weighted least-square criterion,

(5) $$L_{1}(\beta \;j,\lambda_{j})={\left(y_{j}-a_{j}-X_{j}^{T}b_{j}\right)}^{T}Z_{j}\left(y_{j}-a_{j}-X_{j}^{T}b_{j}\right)+\lambda_{1j}\sum_{j^{\prime }=1,j^{\prime }\neq j}^{m}∥\beta_{j|j^{\prime }}{∥}_{2},$$

where **y***_j_ *= (*y_j_*(*W*_1_), …, *y_j_*(*W_n_*)), **μ***_j_ *= (*μ_j_*(*W*_1_), …, *μ_j_*(*W_n_*)), and **b***_j_ *= (**b***_j_*(*W*_1_), …, **b***_j_*(*W_n_*)) determined by **β***_j_* through a nonparametric link; *λ*_1*i*_ is a penalty parameter determined by BIC or extended BIC; and **Z***_j_* = diag{*z_j_*(*W*_1_), …, *z_j_*(*W_n_*)} where *z_j_*(*W_i_*) is a prescribed nonnegative weight function on [*W*_1_, *W_n_*] with boundary conditions *z_j_*(*W*_1_) = *z_j_*(*W_N_*) = 0. This weight function is used to speed up the rate of convergence.

**Adaptive group LASSO**: In group LASSO, penalty parameters of each group are treated equally, without considering the relative importance of different groups. It has been recognized from traditional linear regression analysis that the over-penalization of parameters for some predictors may reduce the efficiency of parameter estimation and the continuity of variable selection.^55^ To overcome this limit of group LASSO, Wang and Leng^56^ integrated it with adaptive LASSO to create adaptive group LASSO. This integrative approach selects significant groups by weighted penalty parameters. Weight *w_j|j_*_′_ is obtained as ${\beta_{j|j^{\prime }}}_{2}^{-1}$ if ${\beta_{j|j^{\prime }}}_{2}$ > 0 and ∞, otherwise. The adaptive group LASSO estimators of dependent parameters are obtained by minimizing the penalized weighted least-square criterion as follow:

(6) $$L_{2}(\beta \;\cdot ,\lambda_{j})={\left(Y_{j}-a_{j}-X_{j}^{T}b_{j}\right)}^{T}Z_{j}\left(Y_{j}-a_{j}-X_{j}^{T}b_{j}\right)+\lambda_{2j}\sum j^{\prime }=1,j^{\prime }\neq jm\;wj|j^{\prime }\prime ∥\beta \;j|j^{\prime }\prime {∥}_{2}$$

where *λ*_2*j*_ is a penalty parameter determined by BIC or extended BIC.

After the most significant links (say *d_j_* ≪ *m*) for each microbe *j* are detected, we substitute them into nLV qdODE in equation (2) to formulate a sparse representation of the full model. The sparse qdODE are written as

(7) $$\frac{dy_{ji}}{dH_{i}}=Q_{j}\left(y_{ji}\left(H_{i}\right);\Theta_{j}\right)+\overset{d_{j}\;}{\underset{j{\;}^{\prime }=1,j^{\prime }\neq j}{\sum }}Q_{jj{\;}^{\prime }}\left(y_{j{\;}^{\prime }i}\left(H_{i}\right);\Theta_{jj{\;}^{\prime }}\right),i=1,\ldots ,n;j=1,\ldots ,m$$

where the notation of the independent and dependent component terms is the same as described in equation (2). By solving equation (7) in which the number of incoming links for a microbe *j* is changed from *m* to *d_j_* through variable selection, we can reconstruct an *m*-node sparse microbial network.

### Reconstructing microbial networks via maximum likelihood

A number of approaches, including non-linear least-squares and maximum likelihood, can be implemented to solve the shrunk qdODE from equation (2). Since we argue that microbial community assembly tends to reach its maximum overall payoff guided by evolutionary game theory, a maximum likelihood approach that is founded on the most probable existence of all microbes is chosen for our qdODE solving. Let $\phi =\left(\mu ;\sum \right)$ denote the parameters that explain the regression model. The likelihood function of ϕ given the abundance data is written as

(8) $$L\left(\mu ;\sum \right)\;=f\left(y_{1},\ldots ,\;y_{m}|\mu_{1},\ldots ,\;\mu_{m}\sum \right)$$

where *f*(⋅) is the *n*-dimensional *m*-variate normal distribution for *m* microbes across *n* samples with mean vector μ and covariance matrix ∑. Specifically, we model the mean vector by equation (7) subject to variable selection, i.e.,

(9) $$\begin{array}{l} \mu =\left(\mu_{1};\ldots ;\mu_{m}\right)=\left(\mu_{1}\left(W_{1}\right),\ldots ,\mu_{1}\left(W_{n}\right);\ldots ;\mu_{m}\left(W_{1}\right),\ldots ,\mu_{m}\left(W_{n}\right)\right)=G_{1}\left(y_{1}\left(W_{n}\right):\Theta_{1}\right)+\sum j\prime =2d_{1}G1j\prime yj\prime \left(W_{n}\right):\Theta 1j\prime ,\ldots ,\;\;G_{m}\left(y_{m}\left(W_{1}\right):\Theta_{m}\right)+\sum j\prime =1d_{m}Gmj\prime yj\prime \left(W_{1}\right):\Theta mj\prime \\ ,\ldots ,G_{m}\left(y_{m}\left(W_{n}\right):\Theta_{m}\right)+\sum j^{\prime }=1,j\prime d_{m}Gmj\prime yj\prime \left(W_{n}\right):\Theta mj \end{array}$$

Now, we implement a power equation-based LOP nonparametric approach to fit $G_{j}\left(\;\right)$ by parameters $\Theta_{j}$ and fit $G_{jj{\;}^{\prime }}\left(\;\right)$ by parameters $\Theta_{jj{\;}^{\prime }}$. We use a statistically robust approach for modeling the covariance matrix,

(10) $$\sum =\left(\begin{array}{ccc} \sum_{1} & \cdots & \sum_{1m}\\ \vdots & \ddots & \vdots \\ \sum_{m1} & \cdots & \sum_{m} \end{array}\right)$$

where $\sum_{j}$ is the sample-dependent covariance matrix of microbe *j*, and $\sum_{j_{1}j_{2}}$ is the sample-dependent covariance matrix between microbes *j*_1_ and *j*_2_. $\sum_{j_{1}j_{2}}$ is structured as $\sum_{j_{1}j_{2}}\sigma_{j_{1}j_{2}}I_{n}$, where $\sigma_{j_{1}j_{2}}$ is the residual covariance of microbes *j*_1_ and *j*_2_ at the same sample. If there is a mix of static and temporal data involved, we may implement the first-order autoregressive (AR(1)) model to model autocorrelative structure of the covariance.

Under mean-covariance structure modeling by equations (9) and (10), model parameters $\phi =\left(\mu ;\sum \right)$ become model parameters $\phi =\Theta_{j},\Theta jj\prime j=1,\ldots ,m,j\prime =1,\ldots ,j-1,j+1,\ldots ,d_{j});$ $\sigma_{j}^{2},\sigma_{j_{1}j_{2}}\left(j_{1}\neq j_{2}=1,\ldots ,m\right)$, whose optimal solution can be obtained, by maximizing the likelihood (8), as

(11) $$\hat{\phi^{\prime }}\in \left\{arg\underset{\phi^{\prime }\in \Phi^{\prime }}{\max}L\left(\phi^{\prime }\right)\right\}$$

Intuitively, this maximization implies an optimal topological structure and organization by which microbes interact with each other to maximize the overall abundance of microbial community assemble as a whole. The convex optimization formulation under equation (11) ensures the stability and sparsity of the network reconstructed from qdODE of equation (7). Since no constraints are given on the number of outgoing links, the resulting network can be high-dimensional and of a large-scale size.

### Topological dissection of microbial networks

Inferred microbial networks via maximizing the likelihood function (8) meet three essential properties of networks, i.e., causality (derived from directed qdODE), sparsity (due to variable selection), and stability (assured under optimization). Apart from the methodological advantages, these networks have many biological merits. First, they are informative because they can capture the full properties of microbial interactions, including *bidirectionality, sign, weight*, and *feedback loop*.^28^ Second, they are dynamically visualizable by reconstructing a series of quasi-dynamic networks along HI gradient from the instantaneous estimates of independent and dependent components for each sample.

Third, the networks reconstructed by qdODE can omnidirectionally capture ecological interactions that occur among microbes. It can cover all possible types of microbial interactions, including *mutualism* (two microbes promote each other by producing factors that are beneficial for both interacting parties), *antagonism* (two microbes inhibit each other), *commensalism* (one microbe promotes its partner whereas the latter does not affect the former), *amensalism* (one microbe inhibits the other and the other is neutral), and *parasitism* (one microbe inhibits the other but the latter promotes the former). The opposite to parasitism is *altruism* (one microbe promotes the other but the latter inhibits the former).^25,49,52^ A microbe may actively manipulate other microbes (by promoting or inhibiting the latter) and, meanwhile, it may be passively manipulated by other microbes. In an idopNetwork, one can identify the numbers of such active links and passive links for each microbe. If a microbe has more active links than passive links, it is regarded as a leader microbe. If a microbe’s active links are more than the average of all microbes (i.e., connectivity), then this microbe is a mighty hub or keystone microbe that is believed to play a pivotal role in maintaining microbial communities. If a microbe has less links, including active and passive, than the average, it is a solitary microbe. The ecological interpretation of these strategies will stimulate researchers to explore the mass, energetic, or signal basis of microbial interactions.^53^

Fourth, taking the means of the estimates of independent and dependent components for a subject from qdODE of equation (7), we can identify a network specifically for this subject. Such personalized microbial networks may provide unique information for designing personalized medicine based on the gut microbiota. Taken together, the qdODE-based networks are informative, dynamic, omnidirectional, and personalized, thus call ideopNetwporks.^28^

### Inferring context-specific microbial networks

In general, gut-microbial studies are designed in a case-control cohort fashion, which allows microbial interactions to be compared between a diseased group and healthy group. This can be achieved by reconstructing context-specific networks. Consider a panel of subjects who are classified into *C* groups or contexts (such as different races, sexes, use vs. no use of a drug, etc.). Here, we formulate a likelihood function which is the same as equation (8), but the mean vector is now modeled as

(12) $$\mu =\left(\mu_{1};\ldots ;\mu_{m}\right)=\left(\mu_{1}\left(W_{1}\right),\ldots ,\mu_{1}\left(W_{n}\right);\ldots ;\mu_{m}\left(W_{1}\right),\ldots ,\mu_{m}\left(W_{n}\right)\right)\;=\sum c=1CG_{1}\left(y_{1}\left(W_{n}\right):\Theta_{1}^{c}\right)+\sum j\prime =2d_{1}G1j\prime yj\prime \left(W_{n}\right):\Theta 1j\prime c,\ldots ,\sum c=1CG_{m}\left(y_{m}\left(W_{1}\right):\Theta_{m}^{c}\right)+\sum j\prime =1d_{m}Gmj\prime yj\prime \left(W_{1}\right):\Theta mj\prime c,\ldots ,\sum c=1CG_{m}\left(y_{m}\left(W_{n}\right):\Theta_{m}^{c}\right)+\sum j\prime =1d_{m}Gmj\prime yj\prime \left(W_{n}\right):\Theta mj\prime c$$

and the covariance matrix modeled similarly as above.

By plugging in the MLEs of mean vectors (9) and (12) into likelihood (8), we obtain the likelihood values L_1_ (assuming that there is no difference among *C* contexts) and L_2_ (assuming that there are differences among *C* contexts), respectively. We further estimate the log-likelihood ratio (LR),

(13) $$LR=-2log\left(L_{0}/L_{1}\right)$$

as a statistic used to test if *n* samples should be sorted into *C* contexts. By reshuffling *n* samples randomly into *C* groups, we calculate the LR value. If this permutation procedure is repeated 1,000 times, we obtain the 95th percentile from 1,000 LR values and use it as a critical threshold.

### Spatial mapping design

#### Patient recruitment and ethics

Six volunteers were recruited for the study at the Department of Gastroenterology and Hepatology of Tianjin Medical University General Hospital. Disease severity in five patients infected with UC was assessed by the modified Mayo endoscopic score. One patient with colonic polyp was marked as a non-UC control (HC). Ethics approval was received from the Tianjin Medical University General Hospital Clinical Research Ethics Committee. All patients signed the informed consent form prior to their operation, and they received polyethylene glycol-based bowel preparation for colonoscopy. Demographic data and clinical characteristics of the UC and non-UC patients are shown in Table 1.

**Table 1.** Disease status and sampling information of UC and non-UC patients.

Patient No.	Sex	Age	UC Stage	Sampling Site	Sampling Approach
1	F	47	Moderate	Cecum Rectum Transverse colon Lumen	BF,DSB,PSB BF,DSB,PSB BF,DSB,PSB
2	M	32	Severe	Cecum Ileum Transverse colon Descending colon	BF,DSB,PSB BF,DSB,PSB BF,DSB,PSB BF,DSB,PSB
3	F	30	Moderate	Rectum Cecum Transverse colon Sigmoid colon Lumen	DSB,PSB DSB,PSB DSB,PSB BF,DSB,PSB
4	F	60	Severe	Cecum Rectum Ileum Transverse colon Lumen	BF BF,DSB,PSB BF,DSB,PSB BF,DSB,PSB
5	M	62	Moderate	Rectum Lumen	DSB,PSB
6	F	47	Non-UC	Cecum Ileum Transverse colon	BF,DSB,PSB BF,DSB,PSB BF,DSB,PSB

BF: biopsy forceps; DSB: disposable specimen brush; PSB: protected specimen brush

DSB used clinically has no plugging structure and is easily disturbed by body fluids or tissues. To address the defects of the existing technology, we developed a PSB as a contrast, which set up a closed plugging device at the end of the DSB seeker using polyethylene glycol. The plugging portion can be tightly integrated with the inner wall of the sheath. The brush was enclosed in the sheath, introduced through the colonoscope channel, and exposed to the external environment when the brush was sent out to the front of the colonoscope. Specific sampling parts were rubbed and rotated around bristles to obtain superficial microbes. After sampling, the brush was retracted into the sheath, then slowly extracted from the colonoscope channel. Samples were immediately placed on liquid nitrogen and stored at – 80°C until DNA extraction.

#### Sample collection and brush sampling

Intestinal sampling involves the lumen and six positions long the gut, ileum, cecum, transverse colon, descending colon, sigmoid colon, and rectum (Table 1). The specimens were sampled from three to four positions in each patient. A total of 23 specimens were collected from four UC patients and one HC.

#### DNA extraction and amplicon sequencing

The QIAamp DNA Mini Kit (Qiagen, Inc, Hilden, Germany) was used to extract DNA from biopsies and brush specimens, according to the manufacturer’s instructions. Primers 515 F and 806 R, each with a barcode and adaptor sequences for high-throughput sequencing, were utilized to amplify the bacterial 16S rRNA gene V4 region; 16S rRNA sequencing was then performed on the Illumina HiSeq2500 platform by Novogene (Beijing, China).

#### Bioinformatics analysis

After removing the barcodes and primers, we merged the reads of each sample using FLASH (version 1.2.7) software to obtain the raw reads.^58^ According to the raw read quality control process of Quantitative Insights Into Microbial Ecology (QIIME, version 1.7.0),^59^ merged raw reads were qualified to generate clean reads: raw reads were cut off at the first base of three or more continuous low-quality bases (the default quality threshold was 19); reads with continuous high-quality bases less than 75% were further filtered out. Chimeras were identified and removed through the UCHIME Algorithm against the Gold database to generate effective reads.^60^ Uparse was used to cluster all effective reads into OTUs with 97% identity;^61^ the highest frequency sequence in each OTU was selected as the representative sequence. The taxonomies were assigned using the Ribosomal Database Project (RDP) classifier^62^ against the Greengenes database (the confidence threshold was 80%), then taxonomic information was obtained and the community composition of each sample was summarized at each level.^63^ The sequences of all samples were normalized based on the sample with the lowest number of sequences (47,986 sequences). The absolute abundance levels of microbes at different taxonomic levels were inferred by taking the product of relative abundance (assessed by 16S rRNA gene amplicon sequencing) and bacterial load at each position (measured by broad-range 16S rRNA gene qPCR).^64,^

## Supplementary Material

Supplemental Material

## Data and code availability statement

The original nucleotide sequences of this study were deposited to the NCBI Sequence Read Archive under accession number SRP128619. The computer code used in this study can be downloaded at https://github.com/CCBBeijing/GutMicrobe or requested from the corresponding author.
