# Supplementary material for: Modeling spatial interaction networks of the gut microbiota
Source: Gut Microbes. 2022 Aug 3;14(1):2106103. doi: 10.1080/19490976.2022.2106103 (PMC9351588; doi:10.1080/19490976.2022.2106103)
Supplement: Supplemental Material [file KGMI_A_2106103_SM4051.docx]

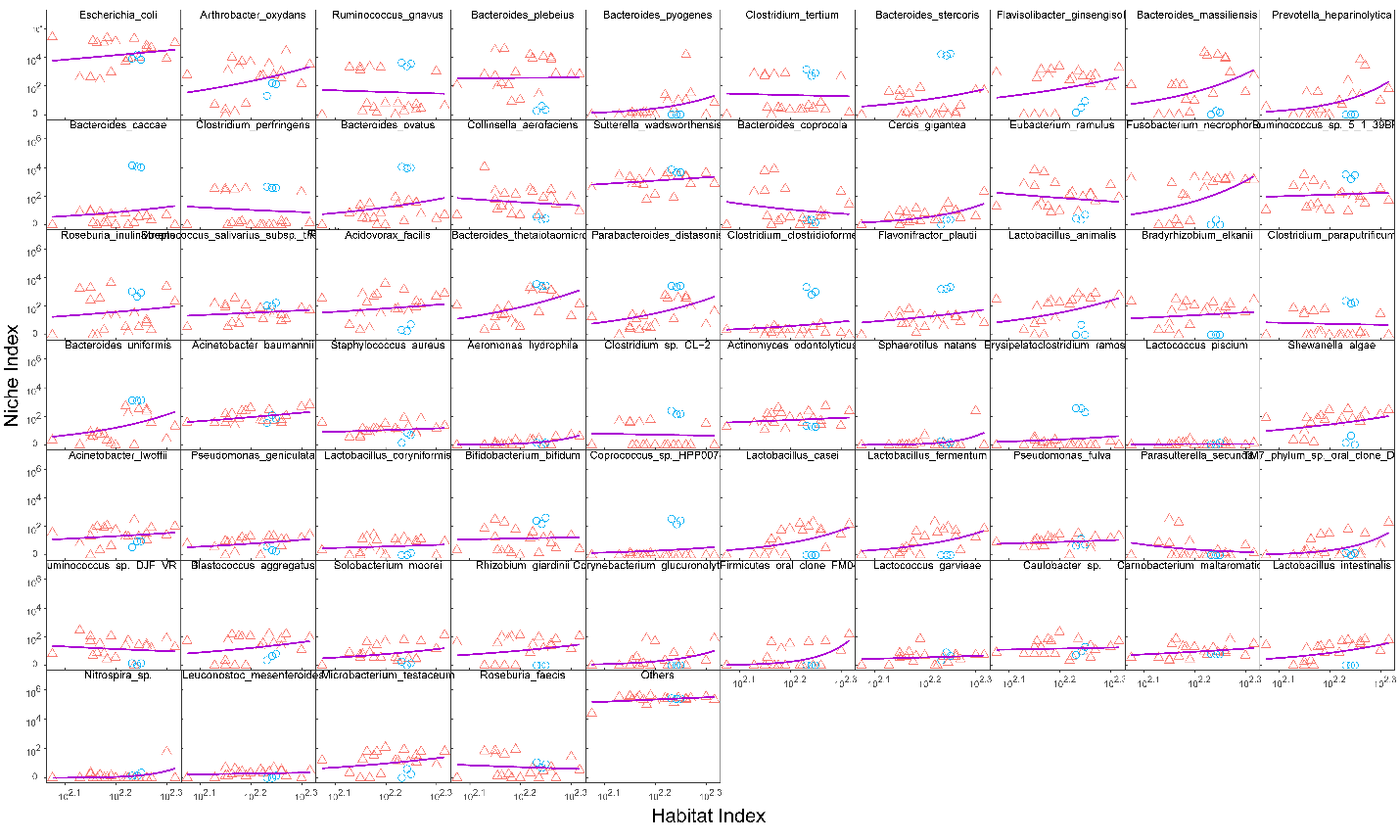


**Figure S1** The fit of the power equation to the relationship between the abundance of individual species (niche index) and the total abundance of all species (habitat index) across UC (red triangles) and HC samples (blue circle).


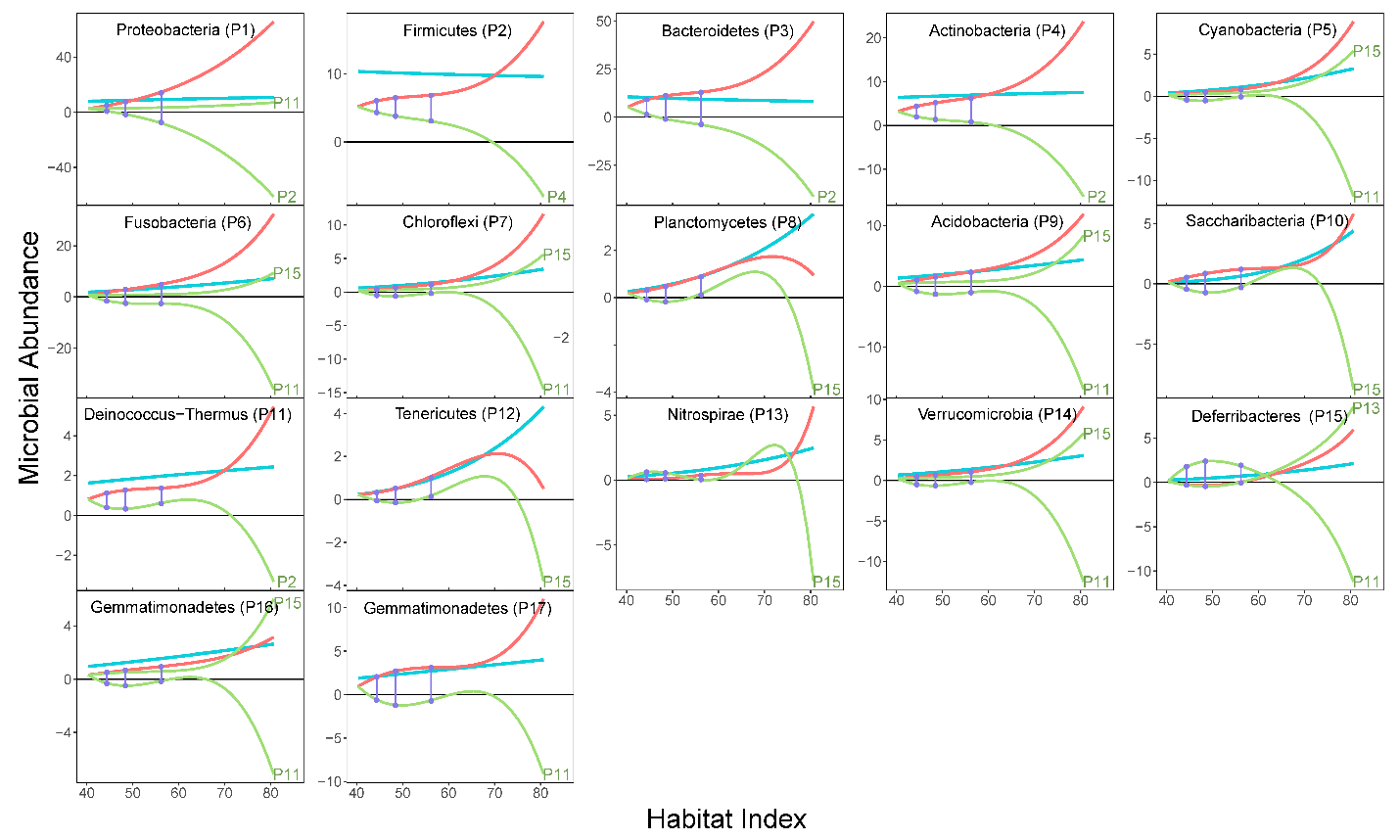


**Figure S2** The decomposition of the net abundance amount of a phylum (P1 – P 17) (blue line) into its independent abundance (red line) arising from the intrinsic capacity of this phylum and dependence abundance (green line) due to the extrinsic influence of other phyla. Note that microbial abundance is expressed as a function of habitat index across samples. Three HC samples are highlighted by linked dots.


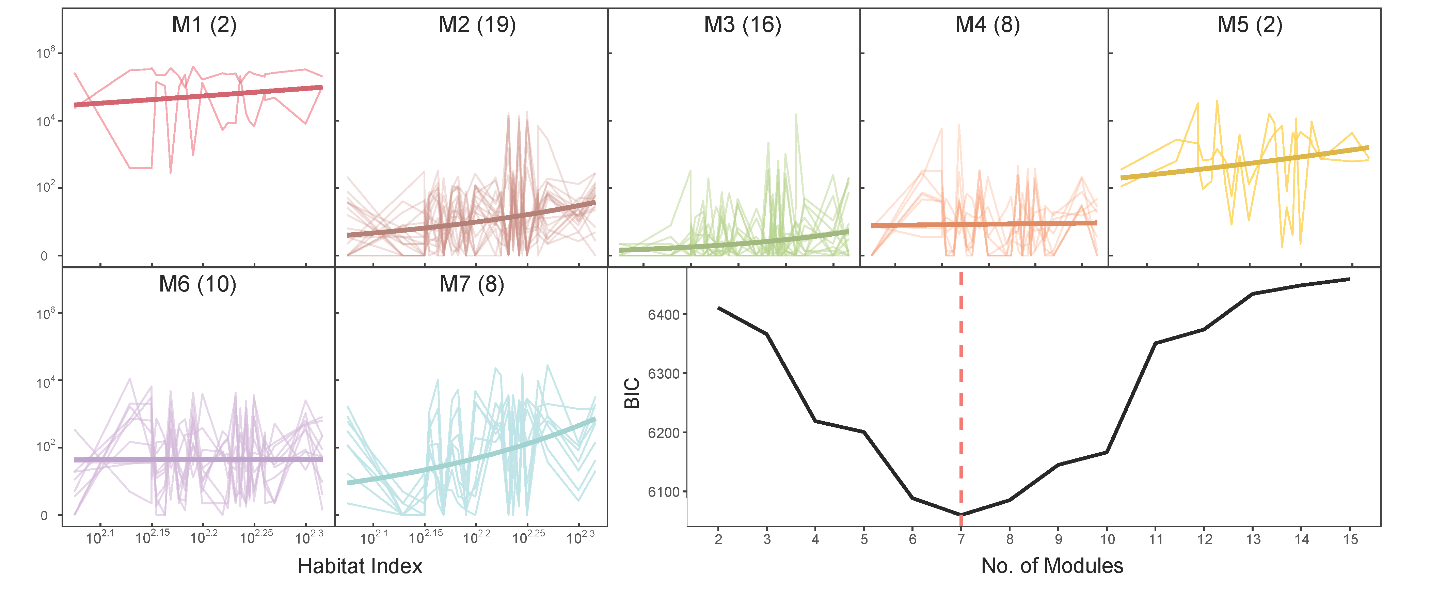


**Figure S3** Functional clustering of 65 bacterial species into eight distinct modules, named M1 – M7, (determined by the BIC plot). The numbers of species within modules are given in parentheses.
